# Public health perinatal promotion during COVID-19 pandemic: a social media analysis

**DOI:** 10.1186/s12889-022-13324-4

**Published:** 2022-05-05

**Authors:** Toluwanimi D. Durowaye, Alexandra R. Rice, Anne T. M. Konkle, Karen P. Phillips

**Affiliations:** 1 Interdisciplinary School of Health Sciences, Faculty of Health Sciences, University of Ottawa, Ottawa, ON K1N 6N5 Canada; 2 University of Ottawa Brain and Mind Research Institute, Ottawa, Ontario Canada

**Keywords:** Social media, Prenatal care, Perinatal care, Pregnancy, COVID-19, Health promotion, Public health

## Abstract

**Background:**

Canadian public health agencies, both municipal/regional and provincial/territorial, are responsible for promoting population health during pregnancy and the early postnatal period. This study examines how these agencies use web-based and Facebook channels to communicate perinatal health promotion during the emergence of the COVID-19 pandemic.

**Methods:**

Perinatal health promotion content of websites and Facebook posts from a multijurisdictional and geographically diverse sample of government and non-governmental organizations (NGO) were evaluated using thematic content analysis in 2020.

**Results:**

Major Facebook perinatal health promotion themes included breastfeeding, infant care, labor/delivery, parenting support and healthy pregnancy. Facebook COVID-19-themed perinatal health promotion peaked in the second quarter of 2020. Websites emphasized COVID-19 transmission routes, disease severity and need for infection control during pregnancy/infant care, whereas Facebook posts focussed on changes to local health services including visitor restrictions. NGO perinatal health promotion reflected organizations’ individual mandates.

**Conclusions:**

Canadian government use of Facebook to disseminate perinatal health promotion during the COVID-19 pandemic varied in terms of breadth of topics and frequency of posts. There were missed opportunities to nuance transmission/severity risks during pregnancy, thereby proactively countering the spread of misinformation.

## Introduction

Perinatal health is informed by the interaction of reproductive determinants, including health services interventions, during the preconception, prenatal and postpartum periods. Pregnancy and the postpartum period are life-stages characterized by rapid change, new routines and experiences. Reproductive decision-making during these periods may include lifestyle changes to mitigate modifiable risk factors, creation of birth plans, selection of prenatal care providers, and choices about breastfeeding, infant care and other postnatal practices [1, 2]. Reproductive health promotion provides information, skills, and resources in preparation for conception, pregnancy, labor and delivery and the postnatal period. Prenatal health promotion, typically emphasizing labor and delivery, often takes the form of prenatal classes, delivered by public health units both in person and online [3]. For those not yet pregnant, a life-course approach to sexual and reproductive health promotion can help mitigate lifestyle risk factors and promote smoking cessation, appropriate gestational weight gain/physical activity and healthy diet in advance of conception [4]. Online prenatal health promotion can bridge gaps in healthcare access, using social media to address emerging health topics, [3] such as Zika virus and the COVID-19 pandemic.

Traditional sources of perinatal and parenting information include healthcare providers, family/friends, and printed media, with Internet/social media and mobile apps common [5–7]. The Internet is a well-established resource for health information related to pregnancy [8–10]. Websites with perinatal health-content are used for health-information-seeking and reproductive-decision-making [5]. Increasingly, local, state/provincial/territorial and federal public health organizations use social media as dissemination channels for health information [11–14]. Advantages of public health messaging via social media include broad population reach, public engagement, opportunities to monitor/correct misinformation, accessibility and timeliness [14].

In Canada, provincial/territorial health ministries along with regional and municipal health authorities are mandated to promote perinatal health in the context of public/population health. A multijurisdictional approach was used to explore website and Facebook-perinatal health messaging by Canadian governmental and non-governmental organizations (NGO) during the emergence of the COVID-19 pandemic in 2020. Facebook is one of the oldest and largest social networking sites, with 83% of Canadian adults active Facebook users, particularly women and adults aged 25–34 years [15]. As adverse pregnancy outcomes are among the sequelae of infectious respiratory diseases, [16] public health perinatal risk messaging specific to COVID-19 was also explored.

## Methodology

### Sample

Purposeful sampling was used to identify Canadian provincial, municipal and NGO, with emphasis on jurisdictional and geographical breadth, and availability of organizational websites (Table 1) and current Facebook pages (Table 2). Urban municipal public health/regional health authorities were geographically distributed and varied in population size [17]. NGO emphasized parenthood/women’s health.

**Table 1:** Organizational Websites Evaluated

Website	Population Size^[17]^/ Jurisdiction
Provincial/Territorial	
Northwest Territories	45,074
Nunavut	39,285
Yukon	42,176
British Columbia (HealthLink BC, Healthy Families BC)	5,145,851
Alberta (MyHealth.Alberta.ca)	4,428,112
Saskatchewan	1,177,884
Manitoba (Healthy Child Manitoba, Manitoba Parent Zone)	1,379,584
Ontario (Ontario Ministry of Children, Community and Social Services, Public Health Ontario)	14,733,119
Quebec (Institut national de santé publique du Québec)^a^	8,575,779
New Brunswick	781,315
Nova Scotia	979,115
Prince Edward Island	159,713
Newfoundland and Labrador	520,998
Municipal	
Whitehorse, Yukon	23,272
Vancouver, British Columbia	600,000
Saskatoon, Saskatchewan	198,958
Winnipeg, Manitoba	632,063
Ottawa, Ontario	812,129
Toronto, Ontario	2,600,000
Montreal, Quebec	1,600,000
Halifax, Nova Scotia	359,111
St. John’s, Newfoundland and Labrador	99,182
Non-Governmental Organizations (NGO)	
Best Start	
The MotHERS Program^b^	
Ontario Prenatal Education	
Dad Central	

^a^Quebec National Institute of Public Health

^b^Mothers’ Health Education, Research & Screening Program

**Table 2:** Facebook Sites Evaluated

Jurisdiction	#Followers^**a**^	#Followers^**b**^/100 K
**Provincial/Territorial**		
Northwest Territories Health and Social Services Authority	1889	4190.9
Yukon Health and Social Services	4979	11,805.3
British Columbia- Provincial Health Services Authority	8796	170.9
Alberta Health Services	87,333	1972.2
Ontario Ministry of Health	149,418	1014.2
Institut national de santé publique du Québec^c^	21,801	254.2
Nova Scotia-Health	45,804	4678.1
Newfoundland- Eastern Health	18,968	3640.7
**Municipal/Regional Health Authority**		
Vancouver Coastal Health	26,789	4464.8
Winnipeg Regional Health Authority	8377	1325.3
Ottawa Public Health- Parenting in Ottawa	20,592	2535.6
Toronto Public Health	29,014	1115.9
CIUSSS de l’Ouest de l’Île de Montréal^d^	14,003	875.2
St. John Public Health- Horizon Health^e^	21,378	2736.2
Halifax- IWK^f^ Health Centre	26,536	7389.4
**NGO**		
Dad Central	1625	–
Native Women’s Association of Canada (NWAC)	49,579	–
The MotHERS Program^g^	569	–
The Society of Obstetricians and Gynaecologists of Canada (SOGC)	22,491	–

^a^Facebook followers represents the number of people who see page updates/posts in their News Feed

^b^Facebook followers represented per 100,000 host region population size (Table 1)

^c^Quebec National Institute of Public Health

^d^Le Centre intégré universitaire de santé et de services sociaux (CIUSSS) de l’Ouest-de-l’Île-de-Montréal- (English: Montréal West Island Integrated University Health and Social Services Centre)

^e^Horizon Health- regional health authority for New Brunswick, Atlantic provinces

^f^IWK- Izaak Walton Killam. ^g^Mothers’ Health Education, Research & Screening Program

### Data extraction

Government/NGO websites were evaluated during the pandemic (August, 2020 to February, 2021). Keyword searches of website homepages, navigation bar documents, dropdown menu articles, and/or PDF documents included terms related to COVID-19, preconception, pregnancy and postpartum stages. Facebook post extraction was performed retrospectively in 2021, using the site search function, logged in from a unique Facebook account to prevent use of cookies/customized search results. Facebook post extraction was limited to 2020, with perinatal keywords reflecting common terms associated with pregnancy, parenthood and newborn care, as follows: pregnancy, fetus, birth, trimester, breastfeed, baby, labour (Canadian spelling), mother, maternity, father, paternity, and postpartum. Extracted search results for analysis included the date, text content and related images for each post [13, 18].

### Data analysis

Websites and Facebook posts were evaluated in a standardized manner using thematic content analysis [19]. Text content related to preconception, pregnancy, and the postpartum period (the first year of life) were inclusion criteria, and subject to analysis. Content/posts with no relevance to perinatal health were excluded from analysis. Themes emerged inductively with emphasis placed on text considered relevant for health promotion purposes, [20] such that websites/Facebook posts often contained multiple themes. A subset of each dataset was evaluated independently by two coders, with consensus meetings used to refine coding if necessary. Facebook post characteristics such as number of posts and temporal trends were evaluated quantitatively [13]. Government website/Facebook content is included here with the original Canadian spelling, punctuation.

## Results

### Perinatal health promotion-Facebook

Individual organizations exhibited considerable variability in the quantity and frequency of perinatal health messaging, quantified by number of relevant Facebook posts in 2020 (Table 3).

**Table 3:** Major Perinatal-Associated Theme

Jurisdiction	#Posts^a^	Major Theme	Post-Day Interval^b^ (mean, SD)
**Provincial/Territorial**			
Northwest Territories Health and Social Services Authority	6	breastfeeding, infant care	44.4 ± 57.82
Yukon Health and Social Services	6	infant care	62.25 ± 75.86
British Columbia- Provincial Health Services Authority	36	COVID-19	10.61 ± 18.08
Alberta Health Services	45	COVID-19, health services	10.78 ± 9.06
Ontario Ministry of Health	8	preterm birth, safety	63 ± 59.65
Institut national de santé publique du Québec^c^	3	infant care, breastfeeding, preterm birth	69.5 ± 4.95
Nova Scotia-Health	20	COVID-19	21.5 ± 18.56
Newfoundland- Eastern Health	34	breastfeeding	10.69 ± 15.43
**Municipal/Regional Health Authority**			
Vancouver Coastal Health	14	COVID-19	25.15 ± 21.57
Winnipeg Regional Health Authority	13	infant care	26.33 ± 28.60
Ottawa Public Health- Parenting in Ottawa	100	infant care	6.16 ± 7.69
Toronto Public Health	24	COVID-19	18.89 ± 21.99
CIUSSS de l’Ouest de l’Île de Montréal^d^	7	breastfeeding, COVID-19	52.83 ± 33.59
St. John Public Health- Horizon Health	30	staff profile	12.69 ± 12.60
Halifax- IWK^e^ Health Center	61	COVID-19	6.75 ± 7.90
**NGO**			
Dad Central	82	fatherhood, parenting support, relationship	4.58 ± 5.09
Native Women’s Association of Canada (NWAC)	16	culture, medical abuse/racism	19.64 ± 22.33
The MotHERS Program^f^	70	healthy pregnancy	5.5 ± 4.14
The Society of Obstetricians and Gynaecologists of Canada (SOGC)	17	labor/delivery	21.19 ± 18.91

^a^Posts with content relevant for perinatal health

^b^Mean days (± standard deviation-SD) since last perinatal-themed post-day

^c^Quebec National Institute of Public Health

^d^Le Centre intégré universitaire de santé et de services sociaux (CIUSSS) de l’Ouest-de-l’Île-de-Montréal- (English: Montréal West Island Integrated University Health and Social Services Centre)

^e^IWK- Izaak Walton Killam.

^f^Mothers’ Health Education, Research & Screening Program

Of the Facebook pages evaluated, Dad Central, the MotHER’s Program, Ottawa Public Health- Parenting in Ottawa, and Halifax IWK Health Center posted the highest number of perinatal health-themed posts in 2020. Facebook perinatal-themed posts generally included perinatal facts/information provided as direct health messaging, or indirect topics for conversation (see below for examples), along with changes to perinatal health services.

**Fig. 1:**
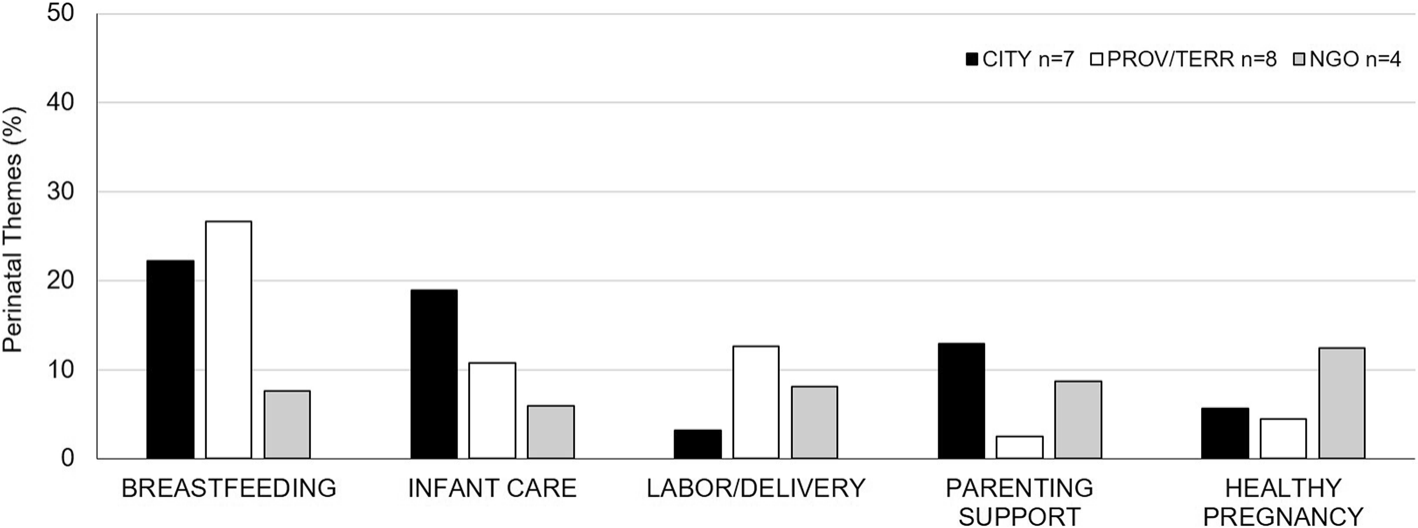
Major Perinatal Themes. For each jurisdiction, major perinatal themes are presented as average themes per Facebook post- see text for post inclusion criteria. CITY- includes both municipal and regional public health authorities - *N* = 249 posts, PROV/TERR- provincial/territorial, *N* = 158 posts, NGO- non-governmental organization, N-185 posts. n- number of unique Facebook sites evaluated per jurisdiction

The most common Facebook perinatal health themes, across all jurisdictions, were breastfeeding, infant care, labor/delivery, parenting support and healthy pregnancy (Fig. 1). Less common perinatal themes included neonatal interventions (18/249 municipal/regional, 8/158 provincial/territory, 1/185 NGO), routine immunizations (19/249 municipal/regional, 6/158 provincial/territory, 0/185 NGO), and lifestyle risks (8/249 municipal/regional, 4/158 provincial/territory, 7/185 NGO).

Most organizations harmonized content and timing of posts with calendar days/weeks recognizing aspects of perinatal health and/or healthcare professionals, such as breastfeeding week or days to recognize health professionals.

“Join the virtual quintessence breastfeeding challenge on Saturday, Oct. 3 as part of breastfeeding week. #LatchOn” Nova Scotia Health, October 1, 2020.

“Today is International Day of the Midwife and we’re celebrating Horizon’s team of registered midwives … Not only is today their day, but the World Health Organization declared 2020 the Year of the Nurse and the Midwife! This small, but mighty, team are primary care providers who train extensively to be experts in healthy and normal pregnancies, labour and births, post-partum, and newborn care.”- New Brunswick- Horizon Health, May 5, 2020.

Another strategy for health promotion was the use of pregnancy-related scenarios or common questions to elicit discussion of perinatal experiences in Facebook comments.

“Good morning! Anyone dealing with nausea during pregnancy? “Morning” sickness is a myth, because it can last ALL DAY!! What tips do you have to help manage nausea?” Parenting in Ottawa, June 28, 2020.

Notifications about upcoming events/prenatal education reinforced and normalized prenatal care and breastfeeding.

“Eastern Health is offering virtual Prenatal Education Classes via Zoom: · Five-week Labour and Birth · Prenatal in a Day · Newborn Care · Breastfeeding · Early Pregnancy · Teen Classes.” -Newfoundland-Eastern Health, September 15, 2020.

Whereas most provincial/territorial and municipal/regional Facebook sites promoted COVID-19 or perinatal health themes (e.g. infant care, breastfeeding), NGO-promoted Facebook topics/themes were influenced by their organizational principles and respective philosophies.

Dad Central, a Canadian NGO that provides networking, resources, training and information in the context of fatherhood, emphasized fatherhood and the importance of supporting one’s partner during parenthood.

“Parents and babies were meant to be connected. Attachment – a strong sense of connection and trust between parent and child – is the foundation of children’s emotional and mental health. That connection also helps you enjoy fatherhood and share the experience of parenting with your partner. That way your baby draws you together and doesn’t pull you apart. It also helps you understand your child, which helps make you a better father. That kind of partner is a mom’s best friend.”- Dad Central, May 11, 2020.

Native Women’s Association of Canada (NWAC), an Indigenous NGO, aims to enhance, promote and foster the social, economic, cultural and political well-being of Indigenous women, girls and gender diverse people within their respective communities and Canadian societies. NWAC’s Facebook posts described Indigenous cultural themes associated with pregnancy and childbirth as well as drawing attention to topical incidents of Canadian healthcare medical abuse/racism experienced by pregnant people.

“Ribbon skirt 1) [name redacted] is an Anishinaabekwe woman from Naongashiing, ON. Her skirt is inspired by a memory of taking a swim with her pregnant sister. ‘The thought of her half submersed in the water with her hair up and her baby bump was breath takingly beautiful. I thought how rich are we to live this life? Surrounded by such land & water, around ceremony & be able to give life: abundance!’”- NWAC, June 5, 2020.

“‘I was thinking my son and I were going to die’. Abuse at Canadian Hospitals is putting Indigenous moms and their babies at risk.” NWAC, November 15, 2020.

### COVID-19-associated perinatal health promotion-websites

**Fig. 2:**
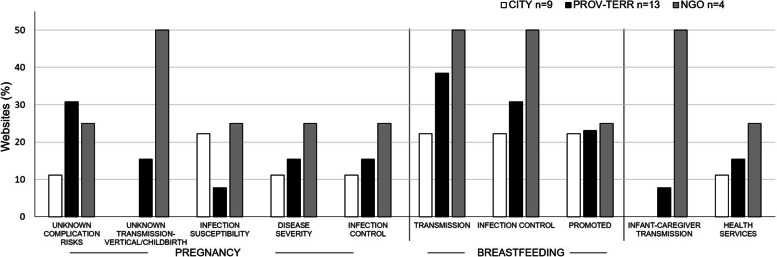
Website Promotion of Perinatal-Associated COVID-19 Information. Major COVID-19-themed perinatal health promotion themes presented per jurisdiction category. CITY- includes both municipal and regional public health authorities. NGO- non-governmental organization

Major perinatal themes from Canadian governmental/NGO website content in 2020 reflected the emergence of COVID-19 and related uncertainty (Fig. 2). Website content was typically limited in both scope and depth in terms of COVID-19-associated perinatal health content, with several websites contributing no relevant content (2/13 provincial-territorial; 7/9 municipal, 2/4 NGO).

Breastfeeding was promoted, even as uncertainties were acknowledged about risks of transmission. Masks and hand hygiene were encouraged during breastfeeding when caregivers were symptomatic or presumptively COVID-19 positive.

“There is limited information about breastfeeding as it relates to COVID-19. In other coronavirus infections, such as Severe Acute Respiratory Syndrome (SARS) and Middle East Respiratory Syndrome (MERS), the virus has not been detected in breastmilk … Breastfeeding is recommended even if you have COVID-19 as there is no evidence that the virus is transmitted in breast milk.”- Toronto.

Similarly, website content recognized evidence gaps:

“Currently, there is no evidence that suggests pregnant women are at a higher risk of getting COVID-19 or if acquired, that they would have a more serious illness. There is also not enough evidence at this time to confirm that a mother can pass COVID-19 to her child during pregnancy.”- Prince Edward Island.

“We do not currently know if pregnant women have a greater chance of getting sick from COVID-19 than the general public nor whether they are more likely to have serious illness as a result … With viruses from the same family as COVID-19, and other viral respiratory infections, such as influenza, women have had a higher risk of developing severe illness.”- Yukon.

Infection control measures including masks, hand hygiene and social distancing were recommended during pregnancy and for caregivers of neonates and infants- recognizing their susceptibility. Regular health services were encouraged to ensure continuity of prenatal care, with the possibility of alternating between in-person and virtual appointments.

As COVID-19 vaccines were approved in Canada in late 2020, some provincial/territorial health (6/13), but no municipal/regional health authority websites provided guidance about vaccines and the implications for pregnancy and breastfeeding.

“The vaccine can be given during pregnancy or breastfeeding if a risk assessment deems that the benefits outweigh the potential risks for the individual and the fetus, and if informed consent includes discussion about the absence of evidence on the use of COVID-19 vaccine in this population. The National Advisory Committee on Immunization has advised that it is prudent to avoid pregnancy for at least 28 days after the second dose of vaccine. Talk to your health care provider for more information.”- New Brunswick.

### COVID-19-associated perinatal health promotion-Facebook

**Fig. 3:**
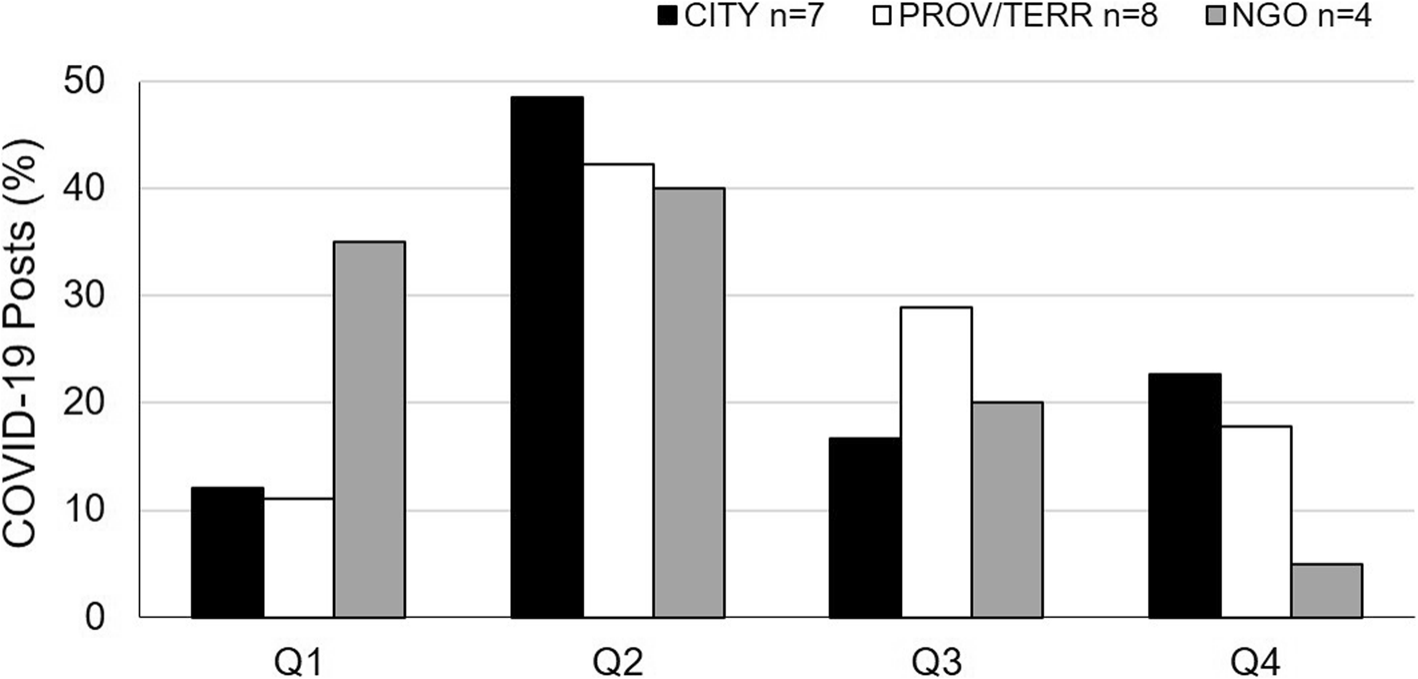
COVID-19 Facebook Posts- 2020. Proportion of COVID-19-themed posts over four quarters of 2020. CITY- includes both municipal and regional public health authorities- *N* = 66 COVID-19-themed posts; PROV/TERR- provincial/territorial *N* = 45 posts; NGO- non-governmental organization; *N* = 20 posts. Q-quarter. n-number of unique Facebook sites per jurisdiction

Across all jurisdictions evaluated, the dominant common Facebook theme associated with perinatal health in 2020 was COVID-19. COVID-19-themed perinatal posts appeared in the first quarter of 2020, consistent with the emergence of the pandemic in Canada in March 2020, peaking in the second quarter (Fig. 3).

**Fig. 4:**
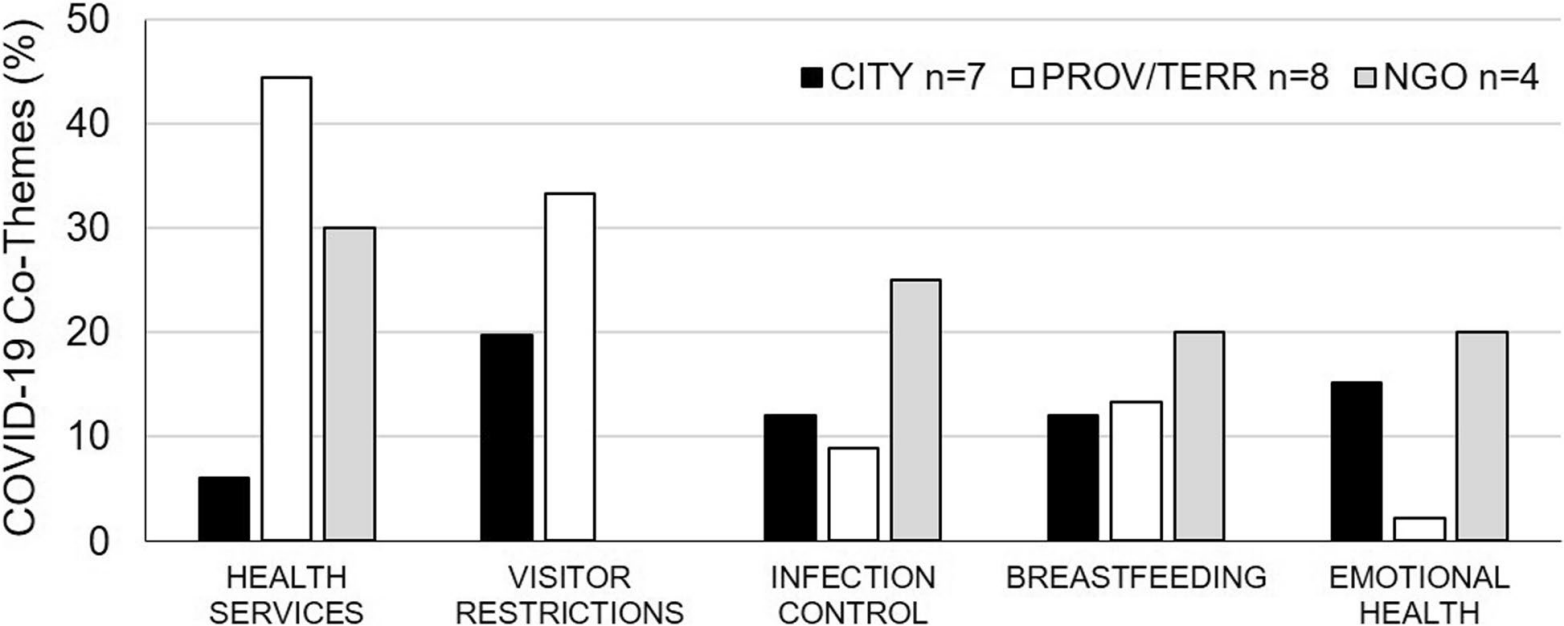
COVID-19-Associated Themes. Major COVID-19-associated themes expressed as jurisdictional average theme per COVID-19 post. CITY- includes both municipal and regional public health authorities- *N =* 66 COVID-19 posts; PROV/TERR- provincial/territorial *N =* 45 posts; NGO- non-governmental organization; *N =* 20 posts. n-number of unique Facebook sites per jurisdiction

In comparison to organizational websites, most of the Facebook sample promoted COVID-19-associated perinatal health content, with the exception of one municipal (1/7) and one NGO (1/4). Provincial/territorial and municipal/regional health organizations posted COVID-19 information primarily in the context of evolving policies around maternal health services including labor/delivery, and related visitor restrictions (Fig. 4).

Infection control- masks, physical distancing and hand hygiene- emerged as a COVID-19-associated theme, primarily in the context of access to health services.

“New mom [name redacted] says that having a baby during the COVID-19 crisis wasn’t much different than when her first child was born. The biggest change is that she had to wear a mask when she was admitted. ‘Breathing through the mask when you’re in labour can be quite hard,’ she says. Regardless, she says she felt cared for and safe during her hospital stay and delivery. ‘If you’re in labour, I think a hospital is the best place to be. You don’t need to be afraid of going,’ she says. ‘There’s hand sanitizer everywhere and the healthcare workers were so caring. You’ll be in the best possible hands, and you’ll get the best possible care.’” -Alberta Health Services, April 29, 2020.

Widespread healthcare visitor restrictions were a common theme, with labor/delivery units consistently limiting visitors to one support companion.

"***PUBLIC SERVICE ANNOUNCEMENT*** Eastern Health Updates on Services and Visitor Restrictions. Eastern Health is advising the public of a reduction of elective and nonurgent services and procedures at Eastern Health facilities across the region … Maternal Fetal Assessment Unit (MFAU) appointments are proceeding and all obstetrics and prenatal appointments as well as high risk pregnancy appointments are going ahead … Please note that the following services will be temporarily interrupted: • All Breastfeeding Support Groups … To limit the spread of respiratory illness, including COVID-19, members of the general public are asked not to visit patients in any hospitals/acute care centres … Only one designated person per patient is permitted in obstetrics delivery rooms." Newfoundland- Eastern Health, March 16, 2020.

Similar to organizational websites, Facebook public health posts recognized that COVID-19 transmission was uncertain but that the benefits of breastfeeding outweighed potential risks.

“COVID-19 has been stressful for a lot of people. Today for #NationalBreastfeedingWeek let’s talk about what it means for new and expectant parents. At this time, there is no evidence that COVID-19 can be passed to the baby through breastfeeding or breast milk. Breastfeeding continues to offer the greatest protection against infection and illness throughout infancy and childhood.” - Northwest Territories Health and Social Services Authority, October 6, 2020.

Finally, the pandemic was recognized as a challenge to emotional health due to both isolation during pregnancy and the early postpartum period, as well as general stress and uncertainty.

“We know that the postpartum time can be challenging for women as they adjust to their new roles, especially for first time mothers. The COVID-19 outbreak has only made getting support and care more difficult and isolating.”- Halifax, IWK Center, August 20, 2020.

## Discussion

Our Internet/social media analysis of perinatal health promotion by a multijurisdictional sample of Canadian governmental/NGO agencies reflected the emergence of COVID-19, impacts on health services and acknowledgement of considerable stress and uncertainty. Although it is well established that infectious respiratory diseases such as SARS, MERS and influenza are associated with increased disease severity and pregnancy complications including preterm birth and spontaneous abortion, [16] Canadian governmental agencies generally missed opportunities to disseminate COVID-19-associated perinatal health information via websites and social media platform-Facebook. Social media posts were regionally-relevant, included local COVID-19 restrictions, reports of outbreaks and evolving hospital/clinic access policies. Breastfeeding, infant care, labor/delivery, parenting support and healthy pregnancy were among the major perinatal themes promoted by governmental Facebook posts. NGO Facebook posts reflected the values and perspectives of host organizations, with Dad Central emphasizing fatherhood and Native Women’s Association of Canada describing cultural and traditional practices associated with pregnancy.

Perinatal health promotion may be addressed in secondary school sexual education, prenatal classes, targeted interventions for communities marginalized by poverty, racism, xenophobia and other structural inequities, and generalized public health messaging. Generalized public health messaging is a cost-effective strategy that can work in concert with individualized counseling, targeted interventions and community-based programs to mitigate modifiable pregnancy risk factors. Such health communication campaigns typically increase uptake of desired health behavior by about 5% [21]. In the context of perinatal health promotion, online public health messaging may reinforce mass media campaigns and social norms about health-risk behaviors such as smoking, drug and alcohol use, and obesity/sedentary behavior, [21, 22] however only two municipal and one provincial government hosted-Facebook posts addressed lifestyle risk factors for perinatal health. As lifestyle risk behaviors are often concordant within couples/families, [23] generalized health promotion targeted to all household members would mitigate pregnancy risks, and increase likelihood of behavior modification.

Major Facebook perinatal-themed posts were positive in tone - emphasizing pregnancy/birth, breastfeeding and parenting. Although all organizations included in our sample promoted perinatal health, their use of Facebook was varied in terms of breadth of topics posted. Breastfeeding was promoted by municipal/regional health both actively (e.g. description of benefits, subject of classes/presentations) and passively (e.g. job titles for profiled staff members, resources). Several organizations used World Breastfeeding Week as a basis to promote breastfeeding, services and staff profiles, previously described as an example of social breastfeeding campaigns [24]. Such promotion campaigns translate to about 18% increase in breastfeeding, proving to be quite effective [21].

### Social media and government

Social media is fairly ubiquitous among commercial organizations, with government agencies slower to capitalize on the benefits of established and emerging media platforms for health promotion[11, 25]. As perinatal health promotion is encompassed within a broader public health mandate by Canadian governments included in our sample, we were unable to assess the general use of social media by these organizations. It was evident that perinatal-themed post frequency varied considerably across jurisdictions, perhaps reflecting only limited use by some agencies. Conceptually, social media health promotion falls within the evolving promise of ‘e-government’ to best use information technologies to enhance activities [26]. The universal reach of Internet- and social media communications in industrialized countries serves to democratize government messaging by widespread accessibility [25, 26]. Governmental public health agencies’ use of social media is primarily unidirectional, used to ensure transparency and accountability through dissemination of organizational updates, health promotion and health policy news [25]. Although we can confirm that Canadian government Facebook posts provided promotion and updates related to perinatal health, we did not assess public response to these posts. Similar to mass media campaigns, [22] Facebook followers (Table 2) receive updated posts with further study required to evaluate awareness, uptake and resulting behavior changes from these perinatal-themed posts.

### COVID-19- crisis communication

Social media’s public reach and timeliness make these platforms ideal for emergency communications [27]. Prior to the emergence of COVID-19, government agencies have used social media for emergency communications related to H1N1, [28] Ebola,[29] Zika virus [30] and a broad spectrum of localized natural disasters and emergencies [27]. As with the Zika outbreak, COVID-19 has serious implications for pregnancy, necessitating perinatal-specific public health guidance [30, 31]. Pregnancy emerged gradually as a risk factor for severe COVID-19 illness [32]. COVID-19 vaccine roll-out across much of the Western world began in late 2020, including Canada, however this was complicated by the initial recommendation by the National Advisory Committee on Immunization (NACI) and other agencies, to withhold COVID-19 vaccination from pregnant people pending further evidence [33]. As our social media analysis was restricted to 2020, we did not capture public health government-Facebook promotion of COVID-19 vaccines, however our review of Canadian government health website content into early 2021 did yield updated NACI-messaging regarding vaccine safety during pregnancy and breastfeeding [34]. Whereas a limited number of government websites addressed COVID-19 risks of pregnancy complications/disease severity, Facebook posts predominantly focussed on disruptions to health services and related visitor restrictions/need for infection control – masks, physical distancing– at healthcare centers.

Government agencies can respond to trending myths or barriers to health behaviors through amplification of evidence-based directives that specifically address rumors and misinformation [27]. Although adverse pregnancy outcomes are now well-established sequelae of COVID-19 [35, 36], throughout 2020, often contradictory public health guidance and misinformation regarding COVID-19 and risks to pregnancy appeared on social media [31]. COVID-19-themed perinatal Facebook posts peaked in the second quarter (April–June) of 2020, with only municipal/regional health authorities exhibiting a modest rebound of activity in the final quarter, indicating missed opportunities by public health agencies to reinforce messaging. Pre-emptive risk communication about COVID-19 vaccines and pregnancy by these public health agencies was warranted, as rumors and misinformation related to infertility, menstrual cycle irregularities and miscarriages accompanied COVID-19 vaccination programs and the eventual prioritization of pregnant people in 2021 [35–37]. Social media provides the opportunity for public health agencies to monitor real-time responses to emergency management and related intervention, but also to curtail the spread of misinformation.

Risk perception is decreased by privilege and social status [38]- such that racialized women- most likely to experience severe COVID-19-related pregnancy outcomes [31]- are also likely to perceive COVID-19 as a high health hazard. In response to the heterogeneous public framing of any health risk issue, influenced by gender, race/ethnicity, emotions and experience, risk communication is most effective if the agency/spokesperson inspires trust [39]. Trust in government agencies may be gained by transparency, credibility, and acknowledgement of scientific uncertainty in the face of evolving information, [27, 39] however countering this trust is social media’s contributions to the increasingly political polarization of science and the recognition of society’s structural racism which contributes to inequities [40]. Performance indicators identified for Canadian public health emergency preparedness included credible, culturally-competent emergency leaders with bilateral crisis communication skills, community engagement, and redundant in emergency communication channels including social media [41]. Ottawa Public Health’s creation of a distinct Parenting in Ottawa Facebook page with 100 perinatal-themed posts in 2020 suggests this agency has fully embraced social media and opportunities of community engagement. Ottawa Public Health consistently attributed authorship of perinatal health Facebook posts to named healthcare professionals with credentials (e.g. registered nurses, lactation consultants, nutritionists)- thereby imbuing their posts with expertise, credibility and authority. Beyond perinatal health, Ottawa Public Health emerged as the most followed local public health unit in North America on Twitter, entertaining and informing over 130,000 followers in 2021 with empathy, honesty, nuance, humor and celebrity endorsements to promote COVID-19 and other public health messaging. Although most public health social media applications emphasize community engagement, social media may also provide platforms for health professionals to network, collaborate and exchange emerging science and practices [42].

### NGO

We included NGO websites and Facebook pages in our analysis, recognizing the diverse landscape of online perinatal health information. NGO content reflected the mission, values and philosophies of each organization, and unlike the governmental organizations in the sample, NGO topic areas were exclusively women, fatherhood and perinatal health. NGO websites more consistently provided COVID-19-themed perinatal health promotion, including content related to disease severity, vertical transmission, breastfeeding and pregnancy outcomes. Similarly, NGO Facebook posts more frequently mentioned infection control, breastfeeding and emotional health, in the context of COVID-19. Two NGOs profile important aspects of perinatal health that are often overlooked- fatherhood and the realities facing Indigenous pregnant people in Canada. Cohabitating fathers and non-biological partners, contribute to both the physical and psychosocial environments of pregnant people. The impact of the non-pregnant partner on pregnancy outcomes has been largely studied heteronormatively, using marriage status among the determinants of concordant health behaviors [43]. Bringing attention to fathers, and non-biological partners recognizes their contributions to modifiable risks to pregnancy such as smoking, exposure to second-hand smoke, physical activity [23] and intimate partner violence and social support [44]. Perhaps due to social support and relationship integrity, fathers’ involvement in pregnancy is associated with better pregnancy outcomes and improved maternal health behaviors [45]. Influenza vaccination exhibits spousal concordance, with female partners generally influenced by positive health behaviors exhibited by male partners, [46] suggesting similar dynamics may occur with COVID-19 protective behaviors, although this remains to be studied.

Indigenous peoples face environmental, economic and health disparities due to colonialism and Canada’s legacy of cultural genocide [47]. Pregnancy outcomes are similarly affected, challenged by limited prenatal and birthing care in rural and remote communities [48]. Recognized as priority communities for public health engagement during emergencies, [41, 49] Indigenous peoples were identified as key populations to receive early COVID-19 vaccinations [50]. Indigenous traditions and stories along with stark depictions of medical abuse/racism comprised the Native Women’s Association of Canada (NWAC)‘s perinatal-themed Facebook posts. The tone of NWAC’s Facebook posts reveals a devastating portrayal of the experience of pregnancy and motherhood in the context of Missing and Murdered Indigenous Women and Girls, and the lasting trauma of forced removal of Indigenous children from their families, first by nationally-run residential schools and now through the child welfare system. Social media and the Internet are useful tools for pregnancy and parenting support, particularly as these modalities enable anonymous information seeking and sharing, thereby reducing discriminatory and stereotyping treatment, and addressing rural and remote barriers to healthcare [51]. Culturally-safe perinatal care, both in-person and through public health Internet and social media messaging, developed collaboratively with Indigenous stakeholders, improves pregnancy and neonatal outcomes [52].

### Limitations

We acknowledge that both government and NGO groups included in our sample provide services and resources addressing a breadth of topics, in addition to the perinatal-themed social media and online content evaluated here. As data collection was completed in early 2021, we also recognize that these organizations may have deleted or modified relevant posts, and may have since updated perinatal promotion messaging with possibly increased or different emphasis. We did not ascertain the public reach of online/social media messaging, nor did we measure Facebook interactions such as post comments, likes or shares. Facebook followers for each page appear in Table 2, which provides a crude estimate of the number of people who viewed each post on their respective Facebook newsfeeds.

## Conclusion

Online/Facebook messaging provides opportunities to reinforce positive perinatal health behaviors and mitigate lifestyle risks such as smoking, obesity and excess gestational weight gain to a wide audience. The pandemic demonstrates the need for both real-time emergency communication but also nuanced discussion about COVID-19 risks to pregnancy, transmission, complications and vaccinations- all of which can be achieved via social media. Use of Facebook for perinatal health promotion was varied among our multijurisdictional, geographically-diverse sample of organizations, with considerable room for improvement in terms of breadth of perinatal-promotion topics, frequency of messaging and COVID-19 information relevant for pregnant people.

## Data Availability

The original Facebook posts evaluated for this study are publicly available using the methodology described herein with keyword searches. Summary data appears within this manuscript. Canadian government/NGO website content has since been updated/archived, with current websites not reflective of our analysis.

## References

[1] Lothian J (2006). Birth plans: the good, the bad, and the future. JOGNN.

[2] Malacrida C, Boulton T (2014). The best laid plans? Women’s choices, expectations and experiences in childbirth. Health (London).

[3] Chedid RA, Terrell RM, Phillips KP (2018). Best practices for online Canadian prenatal health promotion: a public health approach. Women Birth.

[4] Lu MC (2010). We can do better: improving perinatal health in America. J Women's Health (Larchmt).

[5] Lagan BM, Sinclair M, Kernohan WG (2010). Internet use in pregnancy informs women's decision making: a web-based survey. Birth.

[6] Kraschnewski JL, Chuang CH, Poole ES, Peyton T, Blubaugh I, Pauli J (2014). Paging “Dr. Google”: does technology fill the gap created by the prenatal care visit structure? Qualitative focus group study with pregnant women. J Med Internet Res.

[7] Buchanan L, Anderson E, MBiostat HX, Phongsavan P, Rissel C, Wen LM. (2021). Sources of information and the use of mobile applications for health and parenting information during pregnancy: implications for health promotion. Health Informatics J.

[8] Laferriere K, Crighton EJ (2017). “During pregnancy would have been a good time to get that information”: mothers’ concerns and information needs regarding environmental health risks to their children. Int J Health Promot Educ.

[9] Jacobs EJA, van Steijn ME, van Pampus MG (2019). Internet usage of women attempting pregnancy and pregnant women in the Netherlands. Sex Reprod Healthc.

[10] Snyder A, Neufeld HT, Forbes L (2020). A mixed-methods investigation of women's experiences seeking pregnancy-related online nutrition information. BMC Pregnancy Childbirth.

[11] Thackeray R, Neiger BL, Smith AK, Van Wagenen SB (2012). Adoption and use of social media among public health departments. BMC Public Health.

[12] Welch V, Petkovic J, Pardo Pardo J, Rader T, Tugwell P (2016). Interactive social media interventions to promote health equity: an overview of reviews. Health Promot Chronic Dis Prev Can.

[13] Bhattacharya S, Srinivasan P, Polgreen P (2017). Social media engagement analysis of U.S. federal health agencies on Facebook. BMC Med Inform Decis Mak.

[14] Kothari A, Foisey L, Donelle L, Bauer M (2021). How do Canadian public health agencies respond to the COVID-19 emergency using social media: a protocol for a case study using content and sentiment analysis. BMJ Open.

[15] Gruzd A, Mai P. The state of social media in Canada. Ryerson University social media lab Version. 2020;5. 10.5683/SP2/XIW8EW.

[16] Phillips KP, O'Sullivan TL, Dow D, Amaratunga CA (2011). Infectious respiratory disease outbreaks and pregnancy: occupational health and safety concerns of Canadian nurses. Prehosp Disaster Med.

[17] Statistics Canada. Statistics Canada. (2020). Population estimates, quarterly. Table 17-10-0009-01. https://www150.statcan.gc.ca/t1/tbl1/en/tv.action?pid=1710000901

[18] Chou WY, Prestin A, Kunath S (2014). Obesity in social media: a mixed methods analysis. Transl Behav Med.

[19] Vaismoradi M, Jones J, Turunen H, Snelgrove S (2016). Theme development in qualitative content analysis and thematic analysis. J Nurs Educ Pract.

[20] Chedid RA, Phillips KP (2019). Best practices for the design, implementation and evaluation of prenatal health programs. Matern Child Health J.

[21] Snyder LB (2007). Health communication campaigns and their impact on behavior. J Nutr Educ Behav.

[22] Wakefield MA, Loken B, Hornik RC (2010). Use of mass media campaigns to change health behaviour. Lancet.

[23] Shiffman D, Louie JZ, Devlin JJ, Rowland CM, Mora S (2020). Concordance of cardiovascular risk factors and behaviors in a multiethnic US nationwide cohort of married couples and domestic partners. JAMA Netw Open.

[24] Marcon AR, Bieber M, Azad MB (2019). Protecting, promoting, and supporting breastfeeding on Instagram. Matern Child Nutr.

[25] Tursunbayeva A, Franco M, Pagliari C (2017). Use of social media for e-government in the public health sector: a systematic review of published studies. Gov Inf Q.

[26] Small TA (2012). E-government in the age of social media: an analysis of the Canadian government’s use of twitter. Policy Internet.

[27] Khan Y, Tracey S, O'Sullivan T, Gournis E, Johnson I (2019). Retiring the flip phones: exploring social media use for managing public health incidents. Disaster Med Public Health Prep.

[28] Fisher Liu B, Kim S (2011). How organizations framed the 2009 H1N1 pandemic via social and traditional media: implications for U.S. health communicators. Public Relat Rev.

[29] Strekalova YA (2017). Health risk information engagement and amplification on social media. Health Educ Behav.

[30] Lwin MO, Lu J, Sheldenkar A, Schultz PJ (2018). Strategic uses of Facebook in Zika outbreak communication: implications for the crisis and emergency risk communication model Int. J Environ Res Public Health.

[31] McBroom K (2021). A comparison of Zika virus and COVID-19: clinical overview and public health messaging. J Midwifery Womens Health.

[32] CDC. Press Release Thursday, June 25, 2020 CDC updates, expands list of people at risk of severe COVID-19 illness. https://www.cdc.gov/media/releases/2020/p0625-update-expands-covid-19.html

[33] National Advisory Committee on Immunization (NACI) (2020). Archived 2: Recommendations on the use of COVID-19 vaccines.

[34] National Advisory Committee on Immunization (NACI) 2021. Archived 3: Recommendations on the use of COVID-19 vaccines *https://www.canada.ca/en/public-health/services/immunization/national-advisory-committee-on-immunization-naci/recommendations-use-covid-19-vaccines/january-12-2021.html*

[35] SOGC (2021). 2021a SOGC statement on COVID-19 vaccination in pregnancy - revised and reaffirmed November 4.

[36] CDC. New CDC data: COVID-19 vaccination safe for pregnant people. Media Statement For Immediate Release: Wednesday. 2021;(August 11, 2021) https://www.cdc.gov/media/releases/2021/s0811-vaccine-safe-pregnant.html.

[37] SOGC 2021b. Frequently Asked Questions: COVID-19 Vaccine Myths and Facts- September 13, 2021 https://sogc.org/en/-COVID-19/COVID-19/en/content/COVID-19/COVID-19.aspx?hkey=dd7d7494-49fa-4966-ab4d-4dca362a9655

[38] Finucane ML, Slovic P, Mertz CK, Flynn J, Satterfield TA (2000). Gender, race, and perceived risk: the 'white male' effect. Health Risk Soc.

[39] Slovic P (1999). Trust, emotion, sex, politics, and science: surveying the risk-assessment battlefield. Risk Anal.

[40] Ball P, Maxmen A (2020). The epic battle against coronavirus misinformation and conspiracy theories. Nature..

[41] Khan Y, Brown AD, Gagliardi AR, O’Sullivan T, Lacarte S, Henry B (2019). Are we prepared? The development of performance indicators for public health emergency preparedness using a modified Delphi approach. PLoS One.

[42] Eghtesadi M, Florea A (2020). Facebook, Instagram, Reddit and TikTok: a proposal for health authorities to integrate popular social media platforms in contingency planning amid a global pandemic outbreak. Can J Public Health.

[43] Hohmann-Marriott B (2009). The couple context of pregnancy and its effects on prenatal care and birth outcomes. Matern Child Health J.

[44] Shah PS, Shah J (2011). Knowledge synthesis group on determinants of preterm/LBW births. Maternal exposure to domestic violence and pregnancy and birth outcomes: a systematic review and meta-analyses. J Women’s. Health.

[45] Alio AP, Lewis CA, Scarborough K, Harris K, Fiscella K (2013). A community perspective on the role of fathers during pregnancy: a qualitative study. BMC Pregnancy Childbirth.

[46] Cao YJ, Noyes K, Homish GG (2020). Life partner influence on uptake of preventive services: evidence from flu vaccine adoption among the aging population. J Aging Health.

[47] Smylie J, Kirst M, McShane K, Firestone M, Wolfe S, O'Campo P (2016). Understanding the role of indigenous community participation in indigenous prenatal and infant-toddler health promotion programs in Canada: a realist review. Soc Sci Med.

[48] Smylie J, O'Brien K, Beaudoin E, Daoud N, Bourgeois C, George EH, Bebee K, Ryan C (2021). Long-distance travel for birthing among indigenous and non-indigenous pregnant people in Canada. CMAJ..

[49] O'Sullivan TL, Phillips KP (2019). From SARS to pandemic influenza: the framing of high-risk populations. Nat Hazards (Dordr).

[50] Ismail SJ, Zhao L, Tunis MC, Deeks SL, Quach C (2020). National Advisory Committee on immunization. Key populations for early COVID-19 immunization: preliminary guidance for policy. CMAJ..

[51] Wright AL, VanEvery R, Miller V (2021). Indigenous mothers' use of web- and app-based information sources to support healthy parenting and infant health in Canada: interpretive description. JMIR Pediatr Parent.

[52] Brown AE, Middleton PF, Fereday JA, Pincombe JI (2016). Cultural safety and midwifery care for Aboriginal women - a phenomenological study. Women Birth.

